# Radiant Reinforcement: Enhancing Composite Polymer Magnet Materials Mechanical Properties with UVC Medical Disinfection

**DOI:** 10.3390/polym15234551

**Published:** 2023-11-27

**Authors:** Mohamed A. Aboamer, Meshari Algethami, Abdulrahman Hakami, Ahmad Alassaf, Tariq M. Alqahtani, Bakheet Awad Alresheedi, Nader A. Rahman Mohamed

**Affiliations:** 1 Department of Medical Equipment Technology, College of Applied Medical Sciences, Majmaah University, Majmaah 11952, Saudi Arabia; m.aboamer@mu.edu.sa (M.A.A.);; 2 Department of Health Services, Ministry of Defense, Riyadh 56688, Saudi Arabia; 3 Department of Medical Planning, Ministry of Health, Riyadh 11176, Saudi Arabia; 4 Biomedical Engineering Department, Faculty of Engineering, Misr University for Science and Technology (MUST), Giza 12568, Egypt

**Keywords:** UVC irradiation, composite polymer magnet materials, tensile and compression tests, mechanical and chemical properties, medical disinfection

## Abstract

Magnetic polymer composites have recently attracted considerable interest, primarily because of their promising applications, especially in the biomedical industry. The aim of this study is to investigate the impact of ultraviolet C (UVC) irradiation as a disinfection method on the mechanical characteristics of composite polymer magnets. Tensile and compression tests were conducted following the standards set by ASTM D3039 and ASTM D3410, respectively. In addition, energy dispersive spectroscopy (EDS) was used to determine the effect of the disinfection method on the amount of carbon, oxygen, and iron within the surface of the composite polymer magnet material. The UVC’s irradiation impact was statistically assessed by a *t*-test. The results of the tensile tests demonstrated a significant increase in the transition force, measuring 0.41 kN and 0.58 kN before and after UVC exposure, respectively. Similarly, the outcomes of the compression tests showed a notable increase in yield force, registering 4.9 kN and 6 kN before and after UVC treatment. This suggests that the composite magnetic material has gained a higher capacity to withstand compressive loads than tensile loads. Finally, the EDS analysis revealed the carbon mass percentage was 71.69% prior to UVC radiation exposure, with it increasing to 78.56%, following exposure. This suggests that the composite material exhibited improved hardness. These findings highlight that UVC irradiation has a beneficial impact on both the mechanical and chemical properties of the composite magnet material, which support its use as a disinfection method in clinical settings.

## 1. Introduction

### 1.1. Magnetic Polymer Composites

Magnetic polymer composites have recently attracted significant attention due to their potential applications, particularly in the biomedical industry. Among the various areas of interest, three show particular promise: self-healing composites, shape-memory composites, and biodegradable composites. Polymer blends are particularly valuable for applications requiring devices that are mechanically adaptable to cells or various parts of the human body. Furthermore, magnetic polymer composites find utility in all bioMEMS (bio-micro-electro-mechanical system) applications where an external magnetic field can control elements like drug delivery devices, sensors, and motors [1,2,3,4].

The majority of magnetic particles used in the latest iteration of magnetic polymer composites are: (1) neodymium–iron–boron (NdFeB) powdered magnet particles; (2) iron oxide (Fe_3_O_4_); (3) carbonyl iron (Fe(CO)_5_) particles, also known as iron pentacarbonyl, all of which hold great promise for biomedical applications [1,5].

Oxidation and corrosion of polymer-bonded Nd-Fe-B magnets throughout their operational life pose significant challenges. This issue is especially critical as it limits the potential usage of these magnets in demanding conditions such as automotive products, computers, and medical equipment. Several research initiatives by engineers and scientists aim to comprehend the degradation and impact of temperature and humidity on the surface of polymer-bonded Nd-Fe-B magnets [6].

The Crucible Magnetics Division, IG TECHNOLOGIES Inc. (Miami, FL, USA), played a key role in producing magnets with improved resistance to physical deterioration in high-humidity and high-temperature environments (up to 125–150 °C) [7]. Modifying the microstructure of the Nd–Fe–B alloy by introducing cobalt, as discovered by Camp et al., increases the alloy’s resistance to corrosion, as the Nd Co phase is more stable [8].

### 1.2. UV Radiation

UV radiation can be divided into UVA, UVB, and UVC components based on their electrophysical characteristics. UVC photons possess the highest energy and the shortest wavelengths (100–280 nm), while UVA photons exhibit the least energy and the longest wavelengths (315–400 nm). UVB photons lie in between. These UV components can produce a variety of effects on cells, tissues, and molecules [9]. UVC radiation at 254 nm has been demonstrated to effectively eradicate bacteria, viruses, fungi, and even spores [10,11,12]. It achieves this by altering the DNA or RNA structure within microorganisms. Notably, the efficiency varies among different microorganisms due to differences in UV absorption, leading to variable eradication times for each species [13].

### 1.3. Mechanical Test Machine

Mechanical testing machines, often referred to as universal testing machines (UTMs), are sophisticated instruments used to evaluate the mechanical properties of materials. These machines apply controlled forces to test specimens and measure their response, providing crucial data for material characterization, quality control, and engineering design.

Tensile and compression properties continue to play a central role in product design, making tensile and compression testing the most prevalent mechanical tests on diverse materials [10,14,15].

### 1.4. Previous Work

Aleksandar Gruji et al. employed a tensile test to ascertain the mechanical properties of composite polymer magnetic materials with varying mixture ratios. They utilized Nd-Fe-B, barium ferrite, and epoxy resin to create ASTM D3039 tensile samples [16], conducting stress–strain analyses to determine the mechanical traits of the materials. The following three mixing combinations were used: Nd-Fe-B and epoxy resin, barium ferrite and epoxy, Nd-Fe-B, barium ferrite, and epoxy. Notably, the study excluded compression testing and biodegradability assessments based on disinfection methods [17].

Aboamer et al. presented two studies. The first examined the impact of UVC irradiation on the mechanical properties of ABS material, employing three distinct testing methods: tensile, compression, and bending tests. ANOVA analysis determined whether significant differences existed between ultimate stress values in groups subjected to UVC radiation and those that were not. In tensile, compression, and bending tests, the average ultimate stress measured 34.5 MPa, 25.4 MPa, and 24.5 MPa, respectively. UVC radiation exhibited a detectable effect on tensile specimens, with a *p*-value of 0.012, meeting the significance threshold of 0.05 [18]. The second study’s findings revealed that UVB irradiation had a lesser influence on the mechanical properties of low-density polyethylene (LDPE) compared to high-density polyethylene (HDPE). For HDPE’s mechanical tensile qualities, LDPE exhibited less significant influence. Moreover, the *p*-values for yield stress, ultimate stress, and break stress were 3.008 × 10^−4^, 2.5 × 10^−4^, and 0.0075, respectively [19].

### 1.5. Objectives of the Study

The study aims to explore the development of polymeric composite materials integrated with magnetic properties. These novel materials have the potential for diverse applications in the medical field. To assess their mechanical performance in a clinical context, the study aims to design and implement tensile and compression test samples. By subjecting these samples to stress and strain testing both before and after UVC exposure as a medical disinfection process, the research seeks to investigate the influence of the disinfection on their structural integrity. The main goal is to rigorously compare the stress and strain curves from pre- and post-disinfection treatment to determine whether a significant difference exists, providing valuable insights into the suitability of these magnetic polymeric composites for use in medical environments.

## 2. Materials and Methods

**Figure 1:**
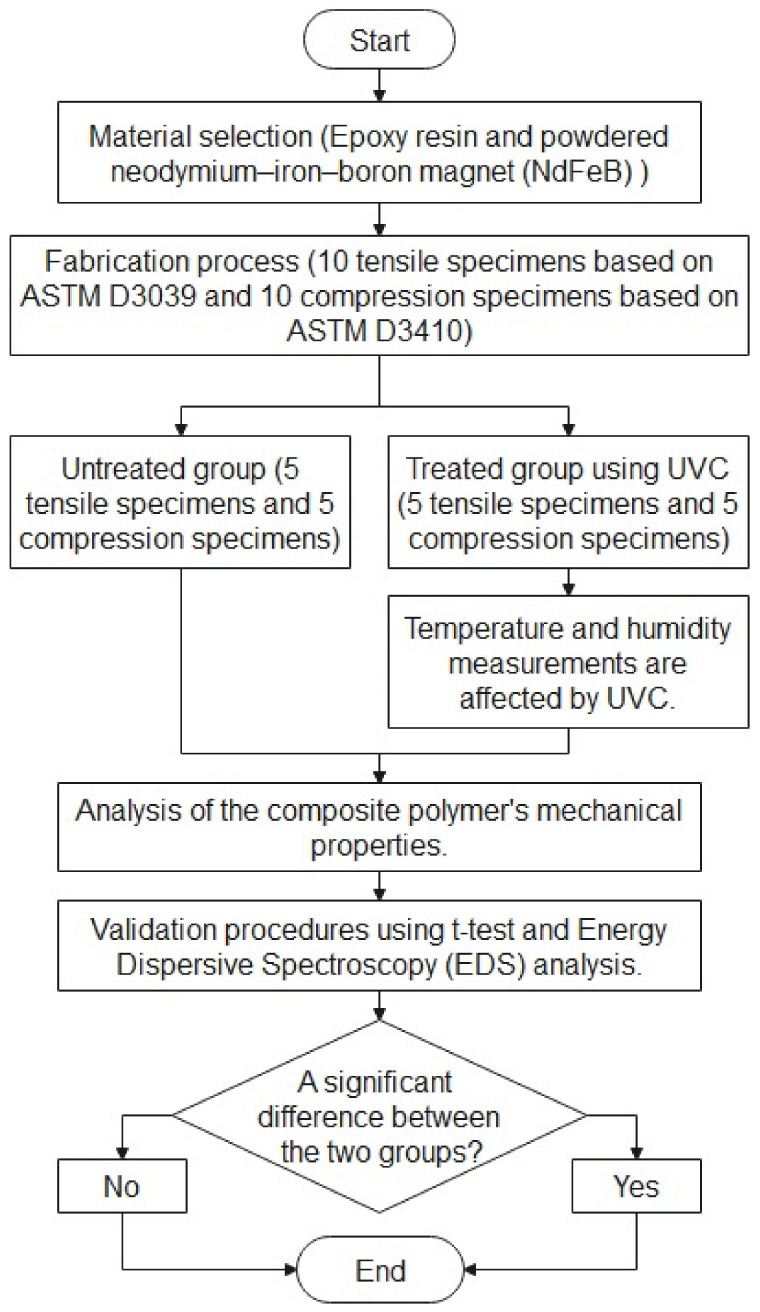
Stages of the proposed method.

The suggested method can be broken down into six distinct stages, as illustrated in Figure 1. These stages are as follows:

1. Material Selection (Stage One): The initial stage involves the careful selection of materials, specifically epoxy resin and powdered neodymium–iron–boron magnet (NdFeB).
2. Manufacturing Procedure (Stage Two): The second step encompasses the manufacturing process, which encompasses the creation of 20 distinct specimens. This batch consists of 10 tensile specimens and 10 compression specimens, adhering to the guidelines provided by ASTM D3039 and ASTM 3410, respectively [16,20].
3. Data Partitioning (Stage Three): Referred to as the third stage, data partitioning involves the separation of specimens into two distinct groups. These are the untreated group, which comprises five tensile and five compression specimens, and the treated group. The treated group also comprises five tensile and five compression specimens that were exposed to ultraviolet light. It is worth noting that the impact of UVC irradiation on temperature and humidity readings is also monitored throughout the treatment process.
4. Examination of Mechanical Characteristics (Stage Four): The fourth stage focuses on a comprehensive examination of the mechanical traits of the composite polymer., by applying controlled forces to all test specimens and measure their response (stress—strain). This data is used to construct a stress–strain curve for the material. Then, key points on the curve, such as the yield point, ultimate strength, and fracture point, are collected to provide valuable information about the material’s mechanical properties and its behavior.
5. Validation Process (Stage Five): The fifth stage entails employing the *t*-test and utilizing Energy Dispersive Spectroscopy (EDS) as a second chemical validation test to determine the effect of the disinfection method on the amount of carbon, oxygen, and iron within the surface of the composite polymer magnet material.
6. Assessment of Group Differences (Stage Six): Lastly, the sixth section revolves around determining whether a significant distinction exists between the two groups.

### 2.1. Material Selection and Specimens Fabrication Process

#### 2.1.1. Silicone Mold

**Figure 2:**
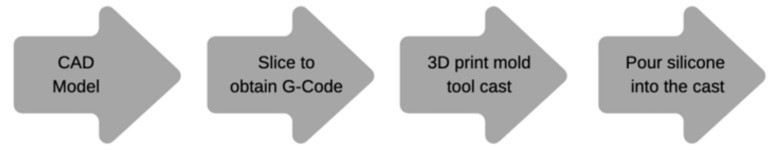
Flow diagram of the mold-making process.

**Figure 3:**
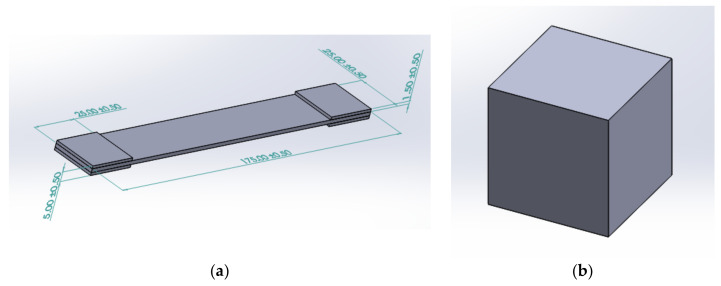
CAD models for tensile and compression tests. (**a**) ASTM D3039 model with full dimensions and (**b**) ASTM D3410 model with 25 mm for each length.

In this investigation, silicone materials were sourced from Castin’ Craft, a company offering materials with a working time of 45 min to one hour and a curing period of 24 h. To provide a visual guide, Figure 2 depicts a flow diagram of the mold-making process. To initiate the creation of a silicone mold tool, the initial step involves designing the mold cast using computer-aided design (CAD) software (SOLIDWORKS Premium 2020 SP0.0). This design incorporates precise measurements, as illustrated in Figure 3. Part (a) corresponds to the ASTM D3039 model for the tensile test, while part (b) represents the ASTM D3410 model for the compression test, each featuring a length of 25 mm.

Upon completing the CAD design, the file is saved in Standard Tessellation Language (STL) format, subsequently transformed into G-Code. Once the G-Code is successfully generated, the mold tool cast can be created using 3D printing technology. For the majority of the project’s tool castings, fused deposition modeling (FDM) was employed. The printing process utilized a Creality Ender 3 printer (Shenzhen Creality 3D Technology Co., Shenzhen, China) and Polylactic acid (PLA) as the chosen printing material. It is worth noting that, given the context, the strength of the mold tool cast is not a primary concern. As a result, a low infill setting of 25% was selected to conserve printing material.

**Figure 4:**
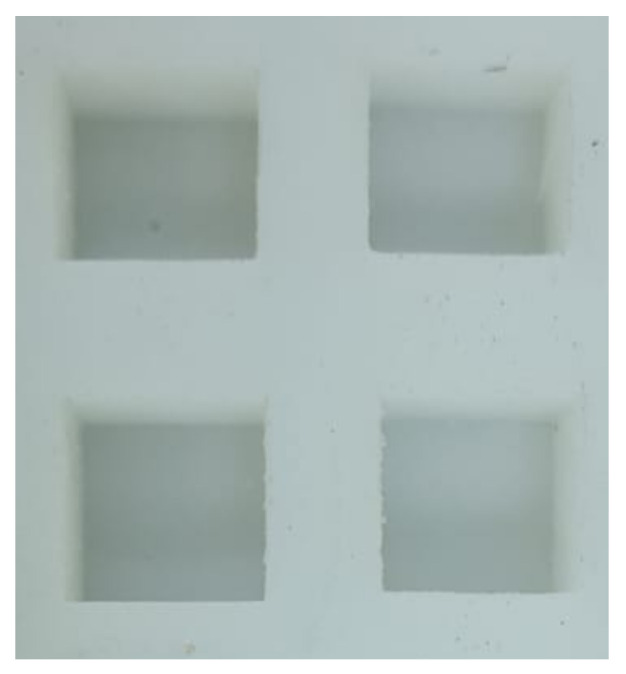
Component finish in silicone mold tool.

Crucially, the component finish should not be overlooked, as it significantly impacts the outcome. The final appearance of the component in the silicone mold tool, as demonstrated in Figure 4, underscores the importance of meticulous consideration for achieving the desired results.

#### 2.1.2. Epoxy Resin

The decision to use epoxy resin as the primary material was grounded in its versatility, cost-effectiveness, and wide availability in the market. The selected epoxy resin, known as Graffiti Resin, possesses the added advantage of being free from volatile organic compounds (VOCs) or, in other words, having zero VOC content. This aligns with the goal of minimizing environmental impact. The mixing protocol for epoxy resin and hardener adheres to the standard practice of a 1:1 ratio, ensuring consistency with established production procedures for epoxy-based products.

Subsequently, the focus shifted to the incorporation of powdered neodymium–iron–boron magnet (NdFeB) into the epoxy resin matrix. This combination was executed at room temperature, facilitating a straightforward and efficient process. The chosen mixing ratio of 50:50 was based on insights gleaned from preceding investigations, serving as a deliberate selection aimed at achieving optimal outcomes.

### 2.2. Geometrical Data of Tensile and Compression Specimens

#### 2.2.1. Tensile Specimens

**Figure 5:**
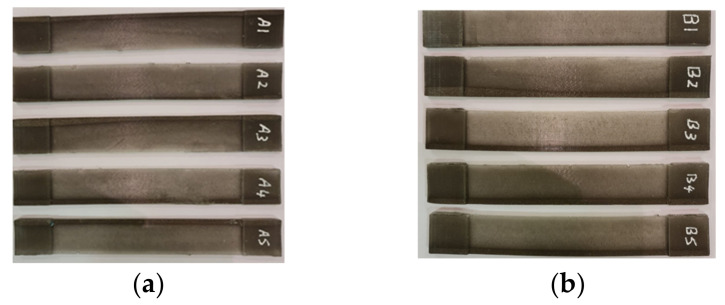
Tensile test specimens (ASTM D3039). (**a**) untreated specimens (A1, A2, A3, A4 and A5) and (**b**) treated specimens with UVC (B1, B2, B3, B4 and B5).

Tensile test specimens were meticulously fabricated in accordance with the rigorous standards outlined by ASTM D3039, all within a controlled ambient temperature of 25 °C. Employing the carefully crafted mold tool, a selection of ten precisely crafted specimens adhering to ASTM D3039 guidelines was executed. These specimens, serving as a foundation for the ensuing evaluations, are visually depicted in Figure 5.

A comprehensive breakdown of the dimensions of these ten ASTM D3039 specimens is thoughtfully presented in Table 1. The entire collection of specimens is effectively divided into two distinct groups, namely the sterilized (B1, B2, B3, B4 and B5) and unsterilized (A1, A2, A3, A4 and A5) categories. The dimensions of each specimen are detailed in the table, encompassing length, width, thickness, and weight, crucial aspects that contribute to the comprehensive understanding of the specimens’ attributes.

The specified dimensions for these specimens, in accordance with ASTM D3039 standards, are as follows: an inner length of 125 mm, an outer length of 175 mm, a width of 25 mm, an outer thickness of 5 mm, and an inner thickness of 1.5 mm. These specific measurements underscore the meticulous adherence to established guidelines, ensuring the reliability and comparability of the ensuing tensile tests.

**Table 1:** Dimensions of ASTM D3039 tensile test specimens (divided into two groups).

Specimen Label	Inner Length [mm]	Outer Length [mm]	Width [mm]	Inner Thickness [mm]	Outer Thickness [mm]	Weight [g]
A1	125.16	174.9	24.9	1.44	5	15.8
A2	125.12	175	24.9	1.42	5.1	15.89
A3	125	174.9	25.1	1.5	5	15.84
A4	124.9	175	25.1	1.4	4.93	15.78
A5	124.9	174.9	24.95	1.44	4.92	15.7
Average	125.02	174.94	24.99	1.44	4.99	15.8
±std	0.12	0.05	0.1	0.04	0.07	0.07
B1	125.1	175	24.98	1.5	4.97	15.89
B2	125.1	175	24.94	1.41	4.99	15.8
B3	125.13	174.9	24.9	1.43	4.95	15.84
B4	124.9	174.9	25.1	1.43	4.97	15.83
B5	124.92	175	24.9	1.44	5	15.81
Average	125.03	174.96	24.96	1.44	4.98	15.83
±std	0.11	0.05	0.08	0.03	0.02	0.04

#### 2.2.2. Compression Specimens

**Figure 6:**
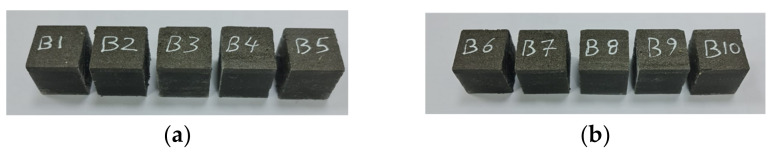
Compression test specimens (ASTM D3410). (**a**) untreated specimens (B1, B2, B3, B4 and B5) and (**b**) treated specimens with UVC (B6, B7, B8, B9 and B10).

Compression test specimens were meticulously crafted in strict accordance with the exacting standards set forth by ASTM D3410. This precision was maintained within a temperature-controlled environment of 25 degrees Celsius. Figure 6 serves as a visual representation of the astute selection of ten meticulously crafted ASTM D3410 specimens, an essential component of the research’s manufacturing process.

Comprehensive insights into the dimensions of these ten ASTM D3410 specimens are systematically provided in Table 2. Each specimen’s characteristics are laid out in detail, encompassing side length and weight in grams. These dimensions collectively contribute to a comprehensive understanding of the specimens’ attributes and characteristics.

The aggregate set of specimens is thoughtfully divided into two distinctive groups: the UVC-treated group (B6, B7, B8. B9 and B10) and the untreated group (B1, B2, B3, B4 and B5). This division underpins the research’s comprehensive evaluation process. The initial column of Table 2 outlines the length of each side in millimeters, while the second column details the weight in grams. These particulars provide vital context for the subsequent compression tests, ensuring an accurate and well-informed analysis.

**Table 2:** Dimensions of ASTM D3410 compression test specimens (divided into two groups).

Specimen Label	Side Length [mm]	Weight [g]
B1	25.1	15.8
B2	25	15.77
B3	25.1	15.8
B4	25	15.77
B5	24.9	15.7
Average	25.02	15.77
±std	0.08	0.04
B6	25	15.76
B7	25.1	15.79
B8	25.1	15.8
B9	25	15.76
B10	25	15.77
Average	25.04	15.78
±std	0.05	0.02

In this experiment, the testing methodology outlined in ASTM D3039 for composite polymer magnets was rigorously adhered to. Specifically, a constant testing speed of 2 mm/min was maintained and data was collected at a frequency of 20 Hz. For the compression test specimen ASTM D3410, the testing speed was set at 1.5 mm/min.

Tensile tests were executed using a universal test machine, equipped with a 5 kN load cell to accurately measure the applied load. The test machine facilitated the recording and preservation of both the tensile load (P) in newtons (N) and the displacement (L) of the upper crossbar in millimeters (mm). This recorded data formed the foundation for the subsequent analyses.

The recorded data underwent a thorough analysis, allowing for the determination of stresses in megapascals (MPa) and non-dimensional strains. This quantitative assessment was achieved through careful computations, enabling the derivation of vital mechanical characteristics, which are subsequently presented as follows:

(1) $$\delta =\frac{P}{W\cdot t}\;\;\;\;\;\;\;\;\;\;\;\;\;\;\;\;\;\;\;\;\;\;\;\;\;\;\;\;\;\;\;\in =\frac{∆L}{L_{0}}$$

where $L_{0}$ is the initial length in millimeters (mm), P is the force in newtons (N), W is the width in millimeters (mm), and t is the thickness in millimeters (mm).

### 2.3. The Role of UV Radiation in the Deactivation of Viruses and Bacteria

Ultraviolet light, a type of electromagnetic radiation, exhibits a wavelength range spanning from 100 to 400 nanometers. Conforming to the specifications of ISO 21348:2007 [21]., ultraviolet radiation can be categorized into four distinct segments: UVA (300–400 nm), UVB (300–280 nm), UVC (200–280 nm), and Vacuum UV–UVV (100–200 nm).

UVA radiation, which falls within the range of 300 to 400 nanometers, is renowned for its role in tanning the skin and its potential to cause long-term damage. Meanwhile, UVB radiation has been harnessed for therapeutic applications, particularly aiding in the management of skin conditions like psoriasis.

On the other hand, UVV radiation’s practicality is limited due to its absorption by the atmosphere, rendering it less relevant in environmental discussions. Of significant note is the unparalleled effectiveness of UVC radiation in combatting microbial threats, including bacteria and viruses [18].

#### 2.3.1. UV Irradiation Enclosure

**Figure 7:**
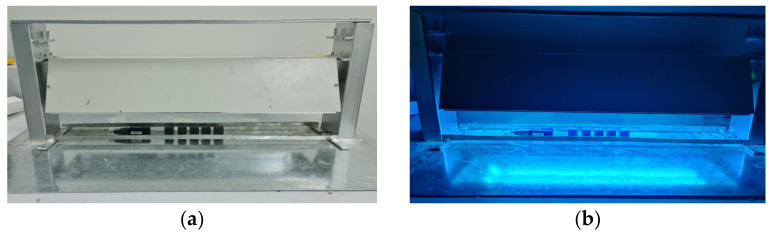
The UV irradiation enclosure. (**a**) the adjustable local made enclosure and the irradiation lamps and (**b**) the arrangement of specimens in the UV radiation enclosure and the wireless data logger.

For the purpose of exposing samples to UV radiation, a custom-built irradiation enclosure was employed. This enclosure was equipped with two 20-Watt UV lamps emitting light at a wavelength of 254 nm. The design of the enclosure allows for flexibility in adjusting the distance between the surfaces of the UV lamps and the surfaces of the samples. Two specific distances were utilized: 8 cm and 16 cm, as depicted in Figure 7a.

The adjustable enclosure, fabricated locally, accommodates the lamps and samples within its structure. The dimensions of the enclosure are 610 × 152 × 108 mm. Additionally, the weight of the enclosure is approximately 4.5 kg [22].

To ensure precise measurement, the irradiance levels within the irradiated region were monitored using a UV radiometer provided by ILT equipment [23]. This instrumentation facilitated accurate tracking of the intensity of UV radiation.

Figure 7b illustrates the arrangement of the specimens within the UV radiation enclosure. During the disinfection period, wireless data loggers were employed to record temperature and humidity measurements [24]. This comprehensive approach to data logging adds an additional layer of insight into the impact of UV radiation on the specimens’ environment.

#### 2.3.2. Exposure Time Calculations

The proposed ultraviolet light-emitting robot featured two UVC lamps, each with a power output of 20 watts. These lamps were positioned at a constant distance of 12 cm from the targeted surfaces. In alignment with findings from earlier research, the requisite dosage for effective inactivation of SARS-CoV-2, the virus responsible for COVID-19, is determined to be 3.7 millijoules per square centimeter (mJ/cm^2^) at a wavelength of 254 nm.

Building on recent investigations [25], it was established that a minimum exposure duration of 30 min was necessary to achieve comprehensive disinfection with a power output of 48 watts.

The following equation can be used to determine the system’s Exposure Duration, which is measured in seconds [26]:

(2) $$Exposure\;Duration\;\left(s\right)=\frac{Dosage\;\left(mJ/{cm}^{2}\right)}{Lamp\;Power\;\left(W/{cm}^{2}\right)}=\frac{2\pi \;LrD}{P}$$

where L is the length of the UVC lamp in centimeters, r is the distance in centimeters, D is the UVC dosage in millijoules per square centimeter, and P is the power of the UVC light that is emitted in watts.

This formula encapsulates a crucial aspect of the research, enabling the determination of the optimal exposure duration essential to achieve the intended level of disinfection. Upon completing the requisite calculations, the essential dose was established at 3.7 mJ/cm^2^—a value pivotal for eradicating SARS-CoV-2, the virus responsible for COVID-19. With the UVC lamps’ dimensions set at 55 cm each, boasting a combined total wattage of 40, and maintaining a separation of 8 cm between the lamps and the surfaces to be disinfected, the calculated exposure time exceeds 48 min. Consequently, for this experiment, a one-hour period was judiciously chosen to facilitate thorough disinfection.

#### 2.3.3. *t*-Test with Two Samples

A *t*-test is a statistical technique that can be employed to compare the means of two distinct groups. The assertion suggesting the absence of a noteworthy distinction between the means of these groups is referred to as the alternative hypothesis. Conversely, the statement indicating a substantial divergence between the means of the groups is recognized as the null hypothesis [27,28,29,30].

The calculation of a two-sample *t*-test involves the utilization of the following equation:

(3) $$t=\frac{\bar{x}-\bar{y}}{\sqrt{\frac{s_{x}^{2}}{n}-\frac{s_{y}^{2}}{m}}}$$

where $\bar{x}$ and $\bar{y}$ represent the means of the samples, $S_{x}$ and $S_{y}$ stand for the standard deviations of the samples, and n and m stand for the sample sizes.

#### 2.3.4. Energy-Dispersive X-ray Spectroscopy (EDS)

Energy-dispersive X-ray spectroscopy (EDS), also referred to as energy dispersive X-ray analysis (EDXA) or energy dispersive X-ray microanalysis (EDXMA), is a widely utilized analytical technique employed to perform elemental analysis or chemical characterization of diverse materials. This method hinges on the interaction that transpires between a source of X-ray excitation and the material under examination.

The energy-dispersive X-ray spectroscopy (EDS) approach facilitates the identification of both major and minor elements, contingent upon their concentrations surpassing 10 wt% (weight percent) for major elements and falling within the range of 1 to 10 wt% for minor elements [30,31]. For this purpose, the Bruker xflash 6130 system (Bruker Company, Billercia, MA, USA), complemented by a cross-sectional scanning electron microscope (SEM) (Nanoscience Instruments, Phoenix, AZ, USA) image at a magnification of 925X, an applied voltage of 20 kV, and a working distance (WD) of 15 mm, was employed to analyze the surface composition of the composite polymer magnet material.

All EDS scanning tests were conducted at the Medical and Scientific Center of Excellence, located in Dokki, Giza Governorate 3750250, Egypt.

## 3. Results

### 3.1. Tensile Test for Untreated Specimens with UV

In this specific experiment, the procedure outlined in ASTM D3039 for testing composite polymer magnets was meticulously followed. Data collection occurred at a frequency of 20 Hz, maintaining a constant speed of 2 mm per minute, as prescribed by the standard.

**Figure 8:**
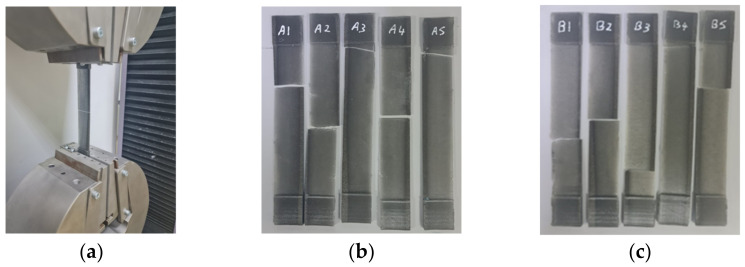
The tensile test. (**a**) Universal testing machine, (**b**) untreated tensile specimens with UVC (A1, A2, A3, A4 and A5), and (**c**) treated tensile specimens with UVC (B1, B2, B3, B4 and B5).

For the mechanical assessment of the material, the ASTM D3039 tensile specimen was affixed to a universal testing machine, as visually represented in Figure 8a. This arrangement enabled the comprehensive exploration of the material’s mechanical properties under controlled conditions.

Then, the impact of UVC treatment on the tensile specimens was evaluated. The initial condition of the tensile specimens prior to UVC treatment is depicted in Figure 8b, whereas Figure 8c showcases the appearance of the same group of tensile specimens’ post UVC treatment. The contrast between these images illustrates the transformative effect of the UVC exposure on the specimens’ physical attributes.

**Figure 9:**
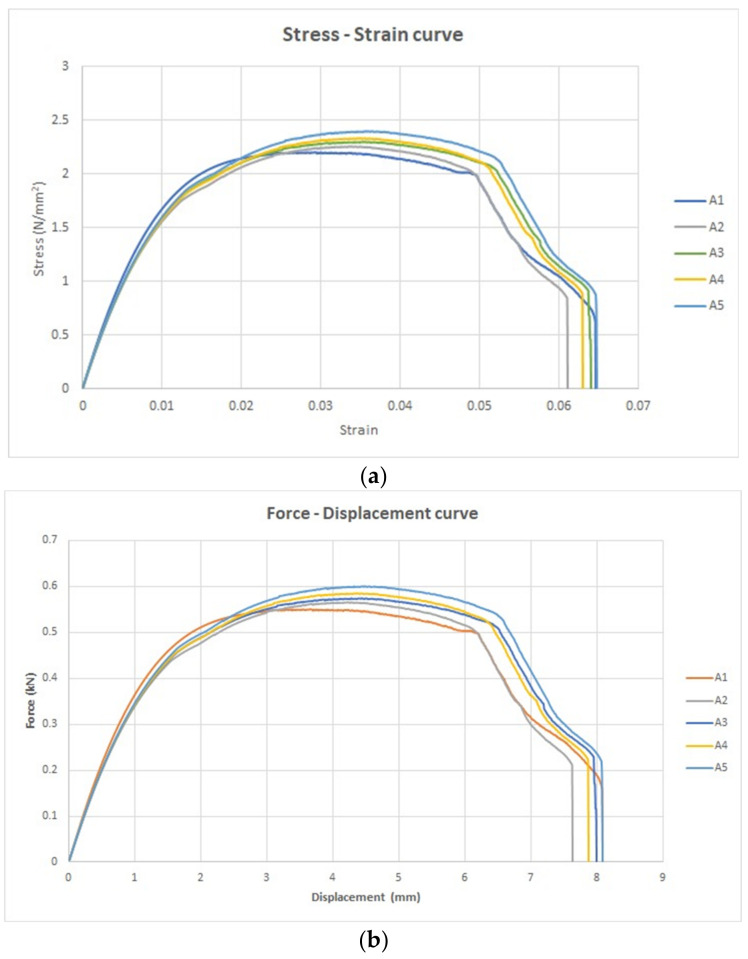
The universal testing machine tensile results. (**a**) The stress strain curves for the untreated five specimens (without UV) and (**b**) the force displacement curves for the untreated five specimens (without UV).

The stress–strain curve is a graphical portrayal of a material’s mechanical characteristics, specifically how it reacts to an externally applied load or stress. Within this context, a group of five tensile specimens was subjected to analysis under the condition titled “without UV.” Stress–strain curves were thereby obtained to explore and understand the material’s mechanical behavior. These five stress–strain curves are collectively illustrated in Figure 9a.

The shape of these stress–strain curves aligns with the curve characterized by a lower slope value in the ASTM D3039 standard. Notably, the stress–strain curve conveys strain along the *x*-axis and stress along the *y*-axis, furnishing insights into the material’s response to varying levels of applied stress.

Furthermore, the force–displacement curve for the same set of five tensile specimens is depicted in Figure 9b. In this representation, the *x*-axis portrays displacement, while the *y*-axis represents force. Crucially, Figure 9a,b emphasize pivotal points on these curves. For Figure 9a, these include:

1. The transition point, marking the boundary between the elastic and plastic regions.
2. The ultimate point, signifying the maximum stress the material can withstand.
3. The fracture point, denoting the conclusion of the plastic deformation region.

Similarly, in Figure 9b, the key points encompass:

1. The transition force, distinguishing the elastic from the plastic domains.
2. The ultimate force, indicating the maximum force the material can endure.
3. The fracture force, highlighting the point of material failure.

The tensile specimens were systematically labeled using letters, with “A” serving as the designation. This labeling scheme corresponds to the specimens’ ordering, with A1 representing the first specimen and A5 signifying the final one, as elucidated in Table 3.

Six distinct parameters were meticulously calculated to accurately encapsulate the material’s mechanical attributes. These parameters include:

1. Transition stress (N/mm^2^)
2. Transition strain
3. Ultimate strain
4. Ultimate stress (N/mm^2^)
5. Fracture strain
6. Fracture stress (N/mm^2^)

The calculated values for these parameters are as follows:

- Average transition stress: 1.6 N/mm^2^
- Average transition strain: 0.03
- Average ultimate strain: 0.03
- Average ultimate stress: 2.3 N/mm^2^
- Average fracture strain: 0.06
- Average fracture stress: 0.71 N/mm^2^

**Table 3:** Mechanical properties of the untreated tensile specimens (without UV).

Specimen Label	Transition Stress (N/mm^2^)	Transition Strain	Ultimate Strain	Ultimate Stress (N/mm^2^)	Fracture Strain	Fracture Stress (N/mm^2^)
A1	1.6	0.09	0.03	2.2	0.07	0.62
A2	1.7	0.01	0.03	2.3	0.06	0.84
A3	1.7	0.01	0.04	2.3	0.06	0.47
A4	1.7	0.01	0.04	2.3	0.06	0.89
A5	1.6	0.01	0.04	2.4	0.07	0.72
Average	1.6	0.03	0.03	2.3	0.06	0.71
±std	0.04	0.04	0	0.08	0	0.17

The concepts of resilience and toughness hold significant relevance within the realm of materials science, particularly in the context of stress–strain curves. In the domain of stress–strain curves, resilience pertains to a material’s capacity to absorb energy without experiencing permanent deformation. In essence, the area beneath the elastic section of the stress–strain curve reflects the material’s resilience. A more resilient material can absorb greater amounts of energy without undergoing lasting deformation or failure.

Conversely, toughness in the context of stress–strain curves signifies a material’s ability to absorb energy without fracturing or breaking. This attribute is quantified by the area beneath the entire stress–strain curve, encompassing the region up to the point of fracture. A higher level of toughness implies that a material can absorb more energy before reaching the breaking point.

As delineated in Table 4, the calculated values for these attributes are as follows:

- Average resilience (energy absorbed in elastic region): 0.01 J/m^3^
- Average toughness (energy absorbed in both elastic and plastic regions): 0.11 J/m^3^.

**Table 4:** Resilience and Toughness for the five untreated tensile specimens (without UV).

Specimen Label	Resilience (J/m^3^)	Toughness (J/m^3^)
A1	0.01	0.11
A2	0.01	0.11
A3	0.01	0.12
A4	0.01	0.12
A5	0.01	0.12
Average	0.01	0.11
±std	0	0.01

The compilation of forces and displacements experienced by the tensile test specimens is succinctly summarized in Table 5. This table encompasses the following parameters:

- Transition force (kN)
- Transition displacement (mm)
- Ultimate displacement (mm)
- Ultimate force (kN)
- Fracture displacement (mm)
- Fracture force (kN)

The computed values for these parameters are as follows:

- Average transition force: 0.41 kN
- Average transition displacement: 0.01 mm
- Average ultimate displacement: 4.2 mm
- Average ultimate force: 0.58 kN
- Average fracture displacement: 7.9 mm
- Average fracture force: 0.18 kN

**Table 5:** Forces and displacements of the untreated tensile specimens (without UV).

Specimen Label	Transition Force (kN)	Transition Displacement (mm)	Ultimate Displacement (mm)	Ultimate Force (kN)	Fracture Displacement (mm)	Fracture Force (kN)
A1	0.4	0.02	3.7	0.55	8.1	0.16
A2	0.41	0	4.2	0.57	7.6	0.21
A3	0.41	0	4.4	0.57	8	0.12
A4	0.43	0	4.4	0.58	8	0.22
A5	0.4	0	4.5	0.6	8.1	0.18
Average	0.41	0.01	4.2	0.57	8	0.18
±std	0.01	0.01	0.34	0.02	0.19	0.04

### 3.2. Tensile Test for Treated Specimens with UV

**Figure 10:**
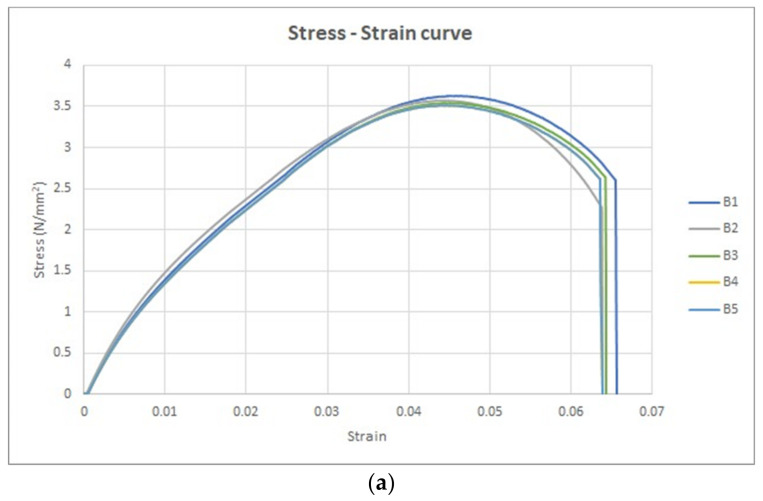

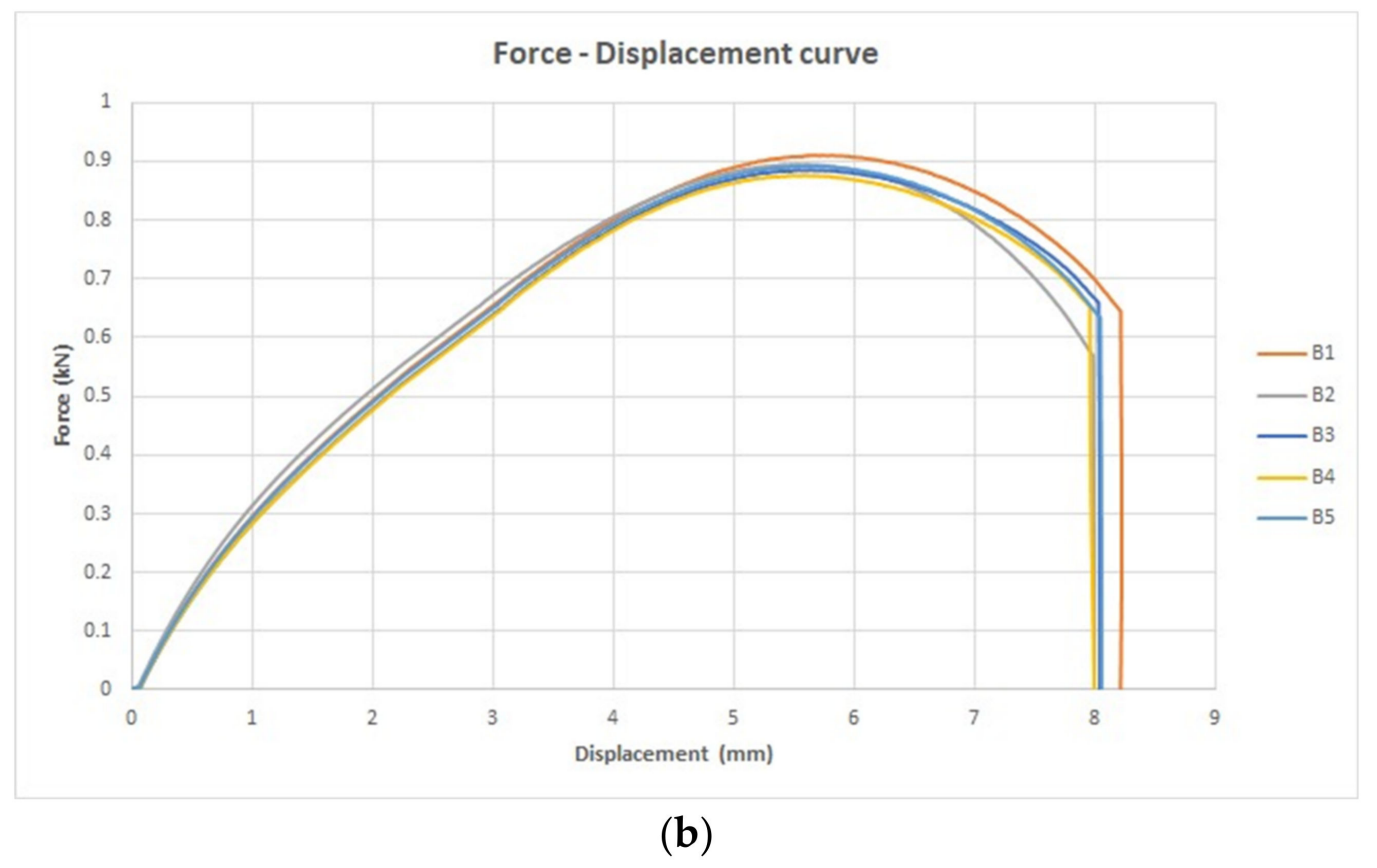
The universal testing machine tensile results. (**a**) The stress strain curves for the treated five specimens (with UV) and (**b**) the force displacement curves for the treated five specimens (with UV).

Upon subjecting the five tensile test specimens to UV irradiation for a duration of 60 min (254 nm wavelength), their mechanical response was altered. Figure 10a visually presents the stress–strain curves generated for these specimens’ post-treatment, offering a clear comparison of their behavior before and after disinfection. Furthermore, the alteration in behavior is similarly captured in Figure 10b, which depicts the force–displacement curves for the same specimens’ post-treatment.

Table 6 comprehensively documents the six pertinent parameters extracted from the post-treatment stress–strain curves. These parameters are classified as follows:

1. Transition stress (N/mm^2^)
2. Transition strain
3. Ultimate strain
4. Ultimate stress (N/mm^2^)
5. Fracture strain
6. Fracture stress (N/mm^2^)

The calculated average values for these parameters following the UV irradiation are as follows:

- Average transition stress: 2.3 N/mm^2^
- Average transition strain: 0.02
- Average ultimate strain: 0.04
- Average ultimate stress: 3.6 N/mm^2^
- Average fracture strain: 0.06
- Average fracture stress: 1.5 N/mm^2^

**Table 6:** Mechanical properties of the treated tensile specimens (with UV).

Specimen Label	Transition Stress (N/mm^2^)	Transition Strain	Ultimate Strain	Ultimate Stress (N/mm^2^)	Fracture Strain	Fracture Stress (N/mm^2^)
B1	2.4	0.02	0.05	3.6	0.07	1.1
B2	2.3	0.02	0.04	3.6	0.06	2.2
B3	2.4	0.02	0.04	3.5	0.06	1.4
B4	2.3	0.02	0.04	3.5	0.06	1.4
B5	2.3	0.02	0.05	3.6	0.06	1
Average	2.3	0.02	0.04	3.6	0.06	1.5
±std	0.06	0	0	0.04	0	0.5

After applying UVC irradiation on the five specimens, the amount of energy absorbed in the elastic or transition region is resilience and the average value of the resilience for the five specimens after being treated by UVC irradiation equals 0.02 J/m^3^. Toughness is the amount of energy absorbed in both the elastic or transition and plastic regions, and the average value of toughness for the five specimens after UVC irradiation is 0.17 J/m^3^ as shown in Table 7.

**Table 7:** Resilience and toughness for the five treated tensile specimens (with UV).

Specimen Label	Resilience (J/m^3^)	Toughness (J/m^3^)
B1	0.02	0.18
B2	0.02	0.17
B3	0.02	0.17
B4	0.02	0.17
B5	0.02	0.17
Average	0.02	0.17
±std	0	0

The forces and displacements experienced by the tensile test specimens are comprehensively summarized in Table 8. This table encompasses a range of crucial parameters, including:

- Transition force (kN)
- Transition displacement (mm)
- Ultimate displacement (mm)
- Ultimate force (kN)
- Fracture displacement (mm)
- Fracture force (kN)

The computed average values for these parameters are as follows:

- Average transition force: 0.58 kN
- Average transition displacement: 2.5 mm
- Average ultimate displacement: 5.5 mm
- Average ultimate force: 0.89 kN
- Average fracture displacement: 6.8 mm
- Average fracture force: 0.36 kN

**Table 8:** Forces and displacements of the treated tensile specimens (with UV).

Specimen Label	Transition Force (kN)	Transition Displacement (mm)	Ultimate Displacement (mm)	Ultimate Force (kN)	Fracture Displacement (mm)	Fracture Force (kN)
B1	0.6	2.6	5.7	0.91	8.2	0.28
B2	0.56	2.3	5.4	0.89	8	0.57
B3	0.59	2.6	5.5	0.89	8	0.36
B4	0.58	2.6	5.5	0.88	8	0.36
B5	0.58	2.6	5.6	0.89	1.6	0.24
Average	0.58	2.5	5.5	0.89	6.8	0.36
±std	0.01	0.16	0.12	0.01	2.8	0.13

### 3.3. t-Test for Tensile Test Groups

**Figure 11:**
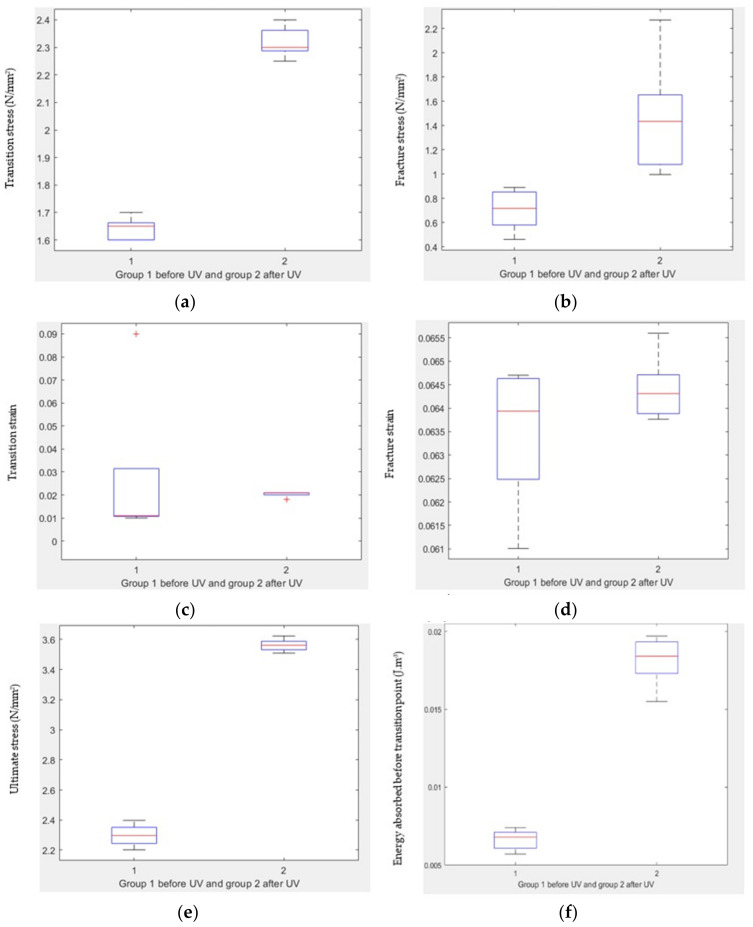

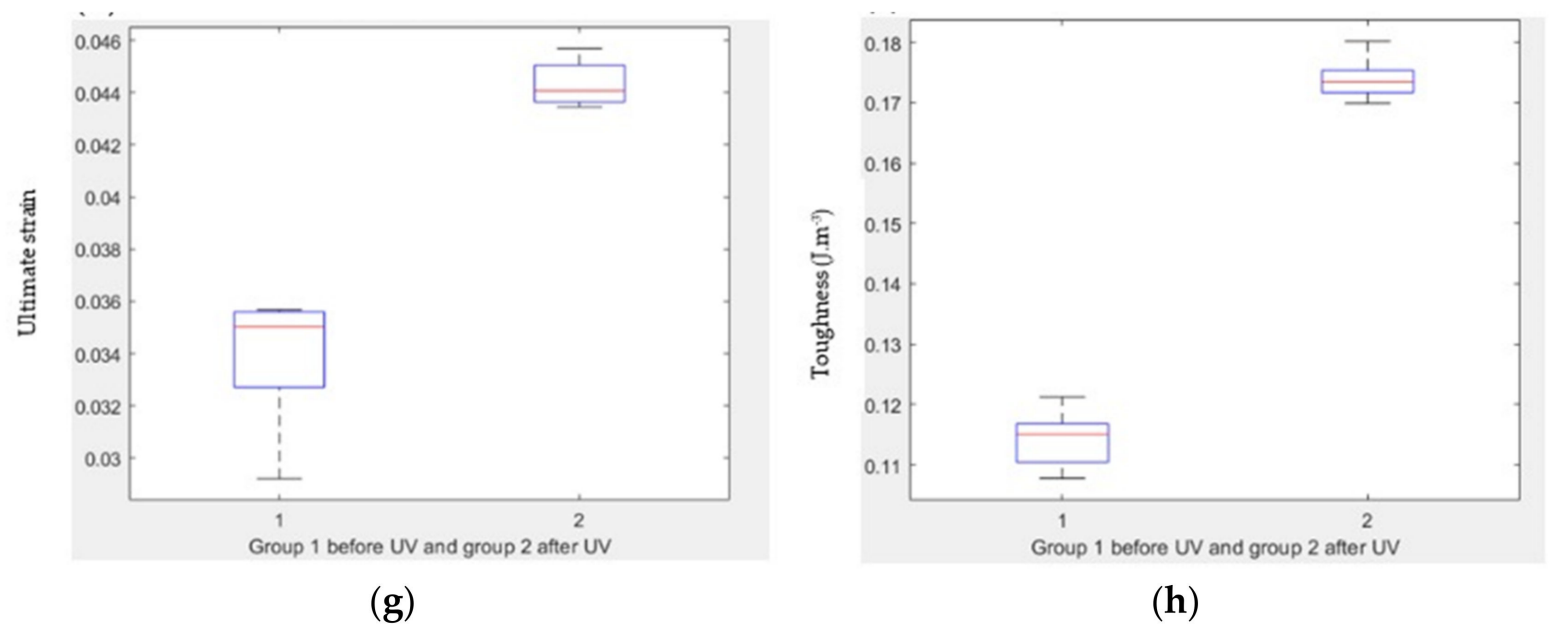
Comparison between treated and untreated samples. (**a**) Boxplot of transition stress before and after UV, (**b**) boxplot of transition strain before and after UV, (**c**) boxplot of ultimate stress before and after UV, (**d**) boxplot of ultimate strain before and after UV, (**e**) boxplot of fracture stress before and after UV, (**f**) boxplot of fracture strain before and after UV, (**g**) boxplot of energy absorbed before transition point before and after UV, and (**h**) boxplot of toughness before and after UV.

Figure 11a illustrates a remarkable distinction in transition stress before and after UV treatment, with a notably low *p*-value of 2.30316 × 10^8^ indicating statistical significance. Upon applying the *t*-test, it was determined that there is no substantial difference in transition strain before and after UV treatment, as evidenced by a *p*-value of 0.6927 (Figure 11b). Similarly, a substantial divergence in ultimate stress before and after UV treatment is demonstrated by a *p*-value of 8.08149 × 10^−10^ (Figure 11c).

For ultimate strain, a boxplot analysis after applying the *t*-test reveals a significant discrepancy before and after UV treatment, with a *p*-value of 3.6569 × 10^−5^ (Figure 11d). Figure 11e underlines a significant difference in fracture stress before and after UV treatment, with a *p*-value of 0.0135. Conversely, no substantial disparity is observed in fracture strain before and after UV treatment, as indicated by a *p*-value of 0.2406 in Figure 11f.

Furthermore, a *t*-test indicates a significant variance in energy absorbed before the transition point before and after UV treatment, evidenced by a *p*-value of 4.76 × 10^−7^ (Figure 11g). Lastly, Figure 11h emphasizes a substantial distinction in toughness before and after UV treatment, denoted by a *p*-value of 2.64 × 10^−8^.

A force displacement curve graphically represents the relationship between the applied force and the resulting displacement or deformation of an object. This curve is a fundamental tool in materials testing and mechanical engineering, providing insights into material behavior under varying loads. The force displacement curve offers a visual representation where the *y*-axis denotes force and the *x*-axis represents displacement or deformation. The curve’s shape varies based on material properties and loading conditions. For instance, in a tensile test on a metal, the curve typically exhibits an initial linear section (elastic region) where the material deforms elastically, followed by a nonlinear segment (plastic region) with permanent deformation before eventual fracture.

**Figure 12:**
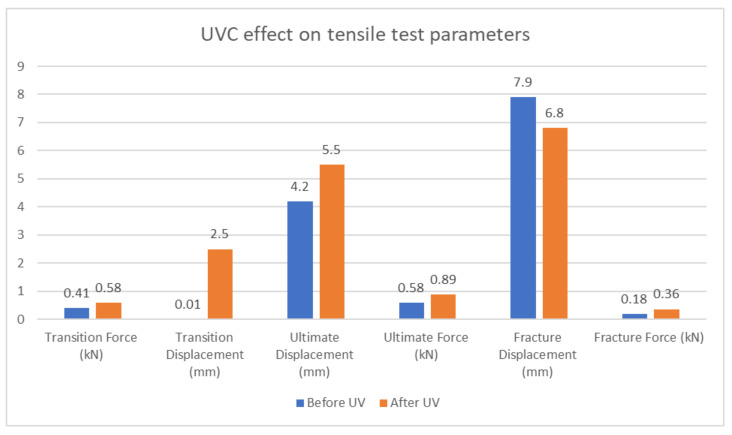
UVC effect on tensile test parameters.

Figure 12 illustrates the force–displacement curve parameters before and after UVC treatment, encompassing transition force, transition displacement, ultimate force, ultimate displacement, fracture force, and fracture displacement. The transition force prior to UVC treatment is 0.41 kN, and it becomes 0.58 kN after treatment. The transition displacement after UVC treatment is 2.5 mm, and the ultimate force before treatment is 0.58 kN, which increases to 0.89 kN post-treatment. Furthermore, the ultimate displacement before treatment is 4.2 mm, which rises to 5.5 mm after UVC exposure. Fracture force prior to UVC treatment is 0.18 kN, changing to 0.36 kN after treatment. Correspondingly, the fracture displacement prior to treatment is 7.9 mm, and post-treatment is 6.8 mm.

The tensile test analysis reveals that there is no noteworthy distinction between the group treated with UVC and the untreated group in terms of transient strain and fracture strain. However, it is evident that the disinfection process using UVC has a noticeable impact on the mechanical properties of the proposed material, composite comprising epoxy, and magnet powder. Specifically, transient stress, ultimate stress, ultimate strain, and fracture stress are notably affected by the UVC disinfection.

Considering these outcomes, it is recommended that future researchers delve into investigating the effects of the disinfection process prior to utilizing the proposed material in medical applications. Understanding how the disinfection process interacts with the material’s mechanical properties is crucial for ensuring its suitability and reliability in medical contexts. This knowledge will contribute to making informed decisions regarding the material’s usage and design, enhancing its performance in medical applications.

### 3.4. Compression Test for Untreated Specimens with UV

**Figure 13:**
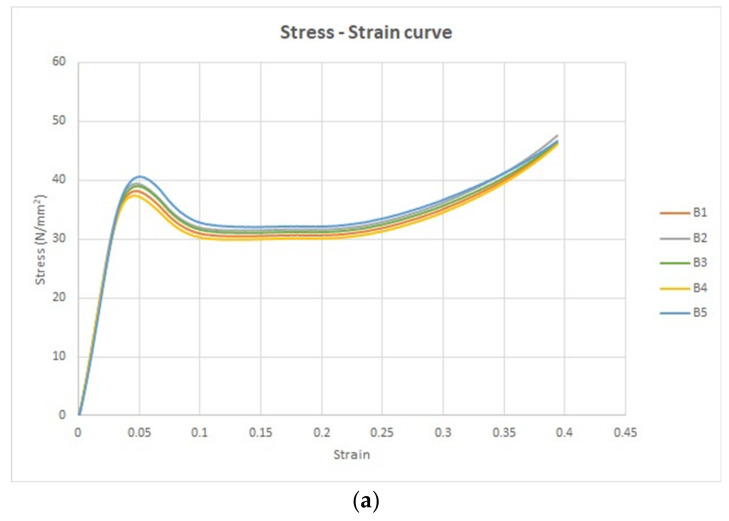

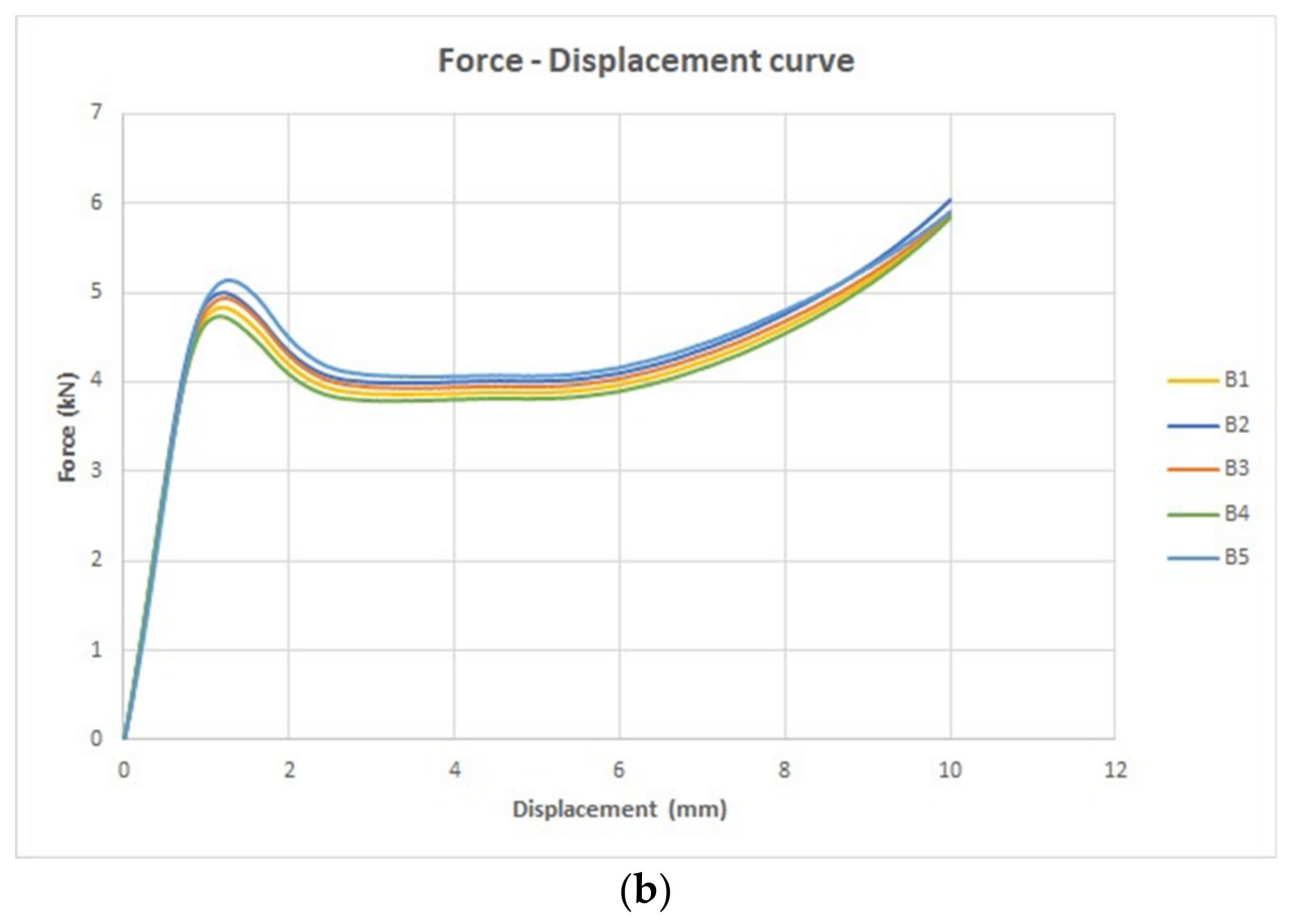
The universal testing machine compression results. (**a**) The stress strain curves for the untreated five specimens (without UV) and (**b**) the force displacement curves for the untreated five specimens (without UV).

Figure 13a illustrates the stress–strain curves of the five compression specimens that have not undergone UVC treatment, providing insight into their mechanical behavior. In Figure 13b, the force–displacement curve for the same untreated UVC compression specimens is depicted, revealing how the applied force relates to the corresponding displacement or deformation. These visual representations contribute to a comprehensive understanding of the material’s response under compression, aiding in the analysis of its mechanical properties.

Upon careful analysis of the stress–strain curves of the five untreated UVC compression specimens, we can extract six crucial mechanical parameters: yield stress (N/mm^2^), yield strain, ultimate stress (N/mm^2^), ultimate strain, fracture stress (N/mm^2^), and fracture strain. Table 9 provides an overview of the average values for these parameters across the five specimens: yield stress averages 38 N/mm^2^, yield strain averages 0.05, ultimate stress averages 38 N/mm^2^, ultimate strain averages 0.05, fracture stress averages 47 N/mm^2^, and fracture strain averages 0.39.

As demonstrated in Table 10, the average absorbed energy within the elastic region of the material is 0.71 J/m^3^, while the calculated toughness is 14 J/m^3^.

Table 11 provides a comprehensive overview of the mechanical parameters extracted from the compression test for the treated UVC compression specimens. These parameters include yield force (kN), yield displacement (mm), ultimate displacement (mm), ultimate force (kN), fracture displacement (mm), and fracture force (kN). The average yield force for the specimens is 4.9 kN, the yield displacement measures 1.2 mm, ultimate force is also 4.9 kN, ultimate displacement averages 1.2 mm, fracture force reaches 5.9 kN, and fracture displacement is 10 mm.

### 3.5. Compression Test for Treated Specimens with UV

**Figure 14:**
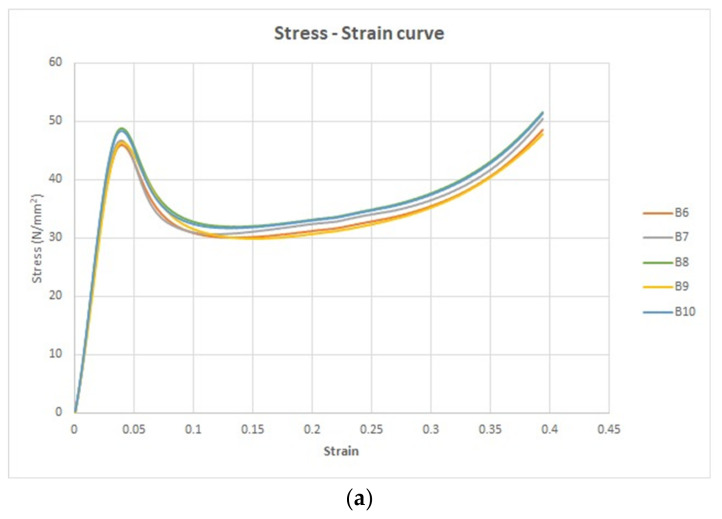

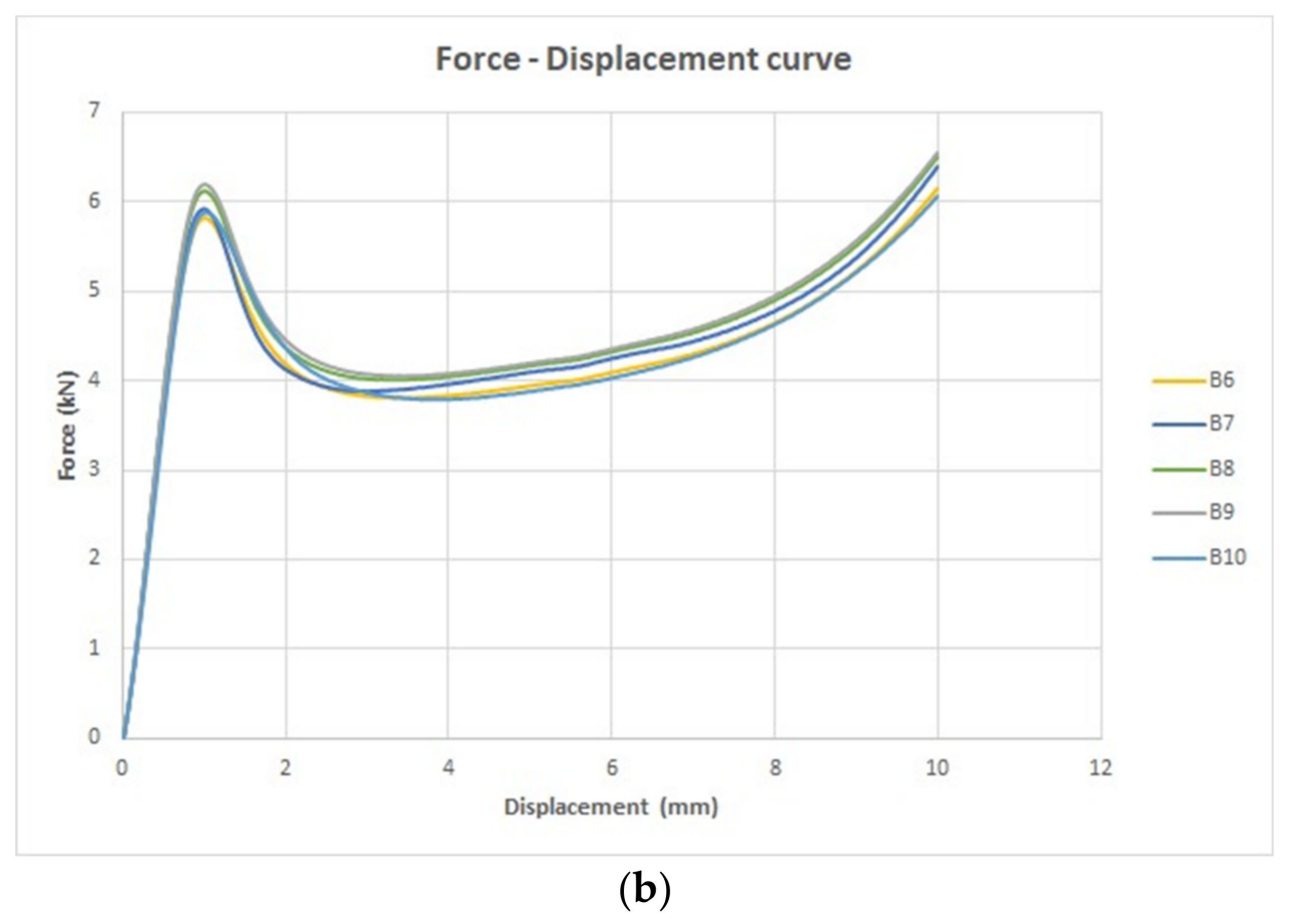
The universal testing machine compression results. (**a**) The stress strain curves for the treated five specimens (with UV) and (**b**) the force displacement curves for the treated five specimens (with UV).

Upon the application of the disinfection process to the five compression test specimens, the resulting stress–strain curves are depicted in Figure 14a. These curves showcase how the material responds to compression forces after undergoing the disinfection treatment. In Figure 14b, the corresponding force–displacement curves are illustrated, shedding light on the relationship between the applied force and the resulting deformation for the treated compression specimens. These visual representations provide valuable insights into how the disinfection process affects the material’s behavior under compression loading conditions.

Table 12 encompasses the critical mechanical properties of the proposed material, featuring six key parameters: yield stress (N/mm^2^), yield strain, Ultimate strain, ultimate stress (N/mm^2^), fracture strain, and fracture stress (N/mm^2^). Across the tested compression specimens, the average values for these parameters are as follows: yield stress averages 47 N/mm^2^, yield strain averages 0.04, ultimate stress averages 47 N/mm^2^, ultimate strain averages 0.04, fracture stress averages 50 N/mm^2^, and fracture stress averages 0.39.

As detailed in Table 13, the computed average value of resilience for the five compression test specimens stands at 0.71 J/m^3^. Additionally, the average toughness of the material is calculated to be 15 J/m^3^.

Table 14 presents a comprehensive overview of key mechanical parameters for the tested compression specimens. The table includes yield force (kN), yield displacement (mm), ultimate displacement (mm), ultimate force (kN), fracture displacement (mm), and fracture force (kN). The computed average values for these parameters are as follows: yield force averages 6 kN, yield displacement averages 1 mm, ultimate force averages 6 kN, ultimate displacement averages 1 mm, fracture force averages 6.3 kN, and fracture displacement averages 10 mm.

### 3.6. t-Test for Compression Test Groups

**Figure 15:**
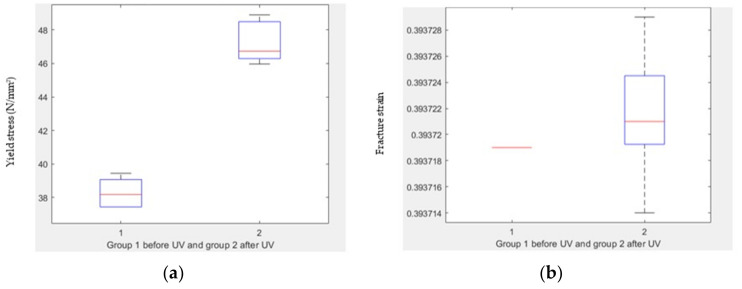

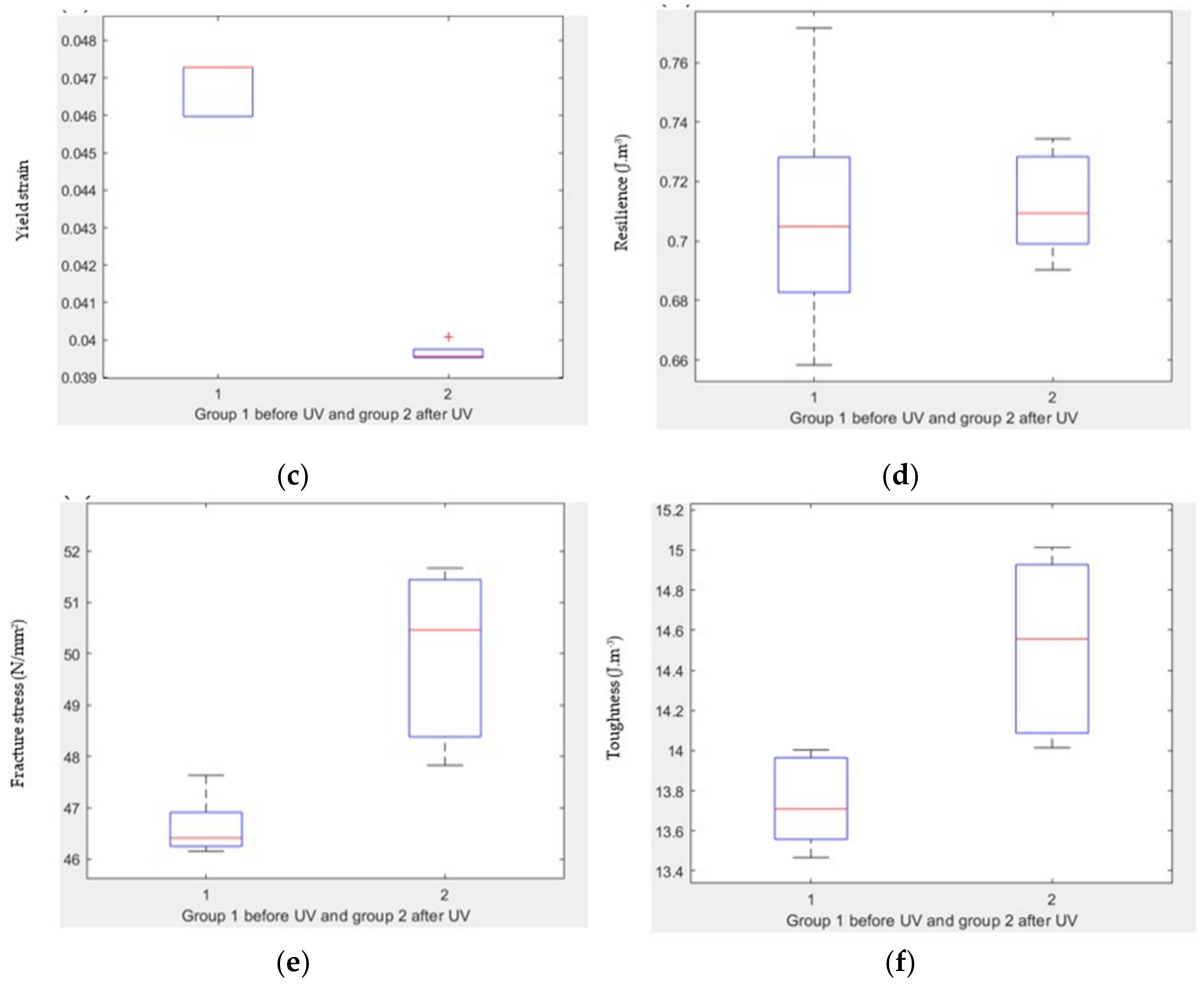
Comparison between treated and untreated samples. (**a**) Boxplot of yield stress before and after UV, (**b**) boxplot of yield strain before and after UV, (**c**) boxplot of fracture stress before and after UV, (**d**) boxplot of fracture strain before and after UV, (**e**) boxplot of resilience before and after UV, and (**f**) boxplot of toughness before and after UV.

The results of the statistical analysis show the impact of UV treatment on various mechanical properties of the material. Here are the findings for different parameters along with their associated *p*-values:

- Yield stress: There is a significant difference in yield stress before and after UV treatment, with a *p*-value of 1.29 × 10^−6^ (Figure 15a).
- Yield strain: There is a significant difference in yield strain before and after UV treatment, with a *p*-value of 2.78 × 10^−8^ (Figure 15b).
- Fracture stress: There is a significant difference in fracture stress before and after UV treatment, with a *p*-value of 0.0032 (Figure 15c).
- Fracture strain: There is no significant difference in fracture strain before and after UV treatment, with a *p*-value of 0.3102 (Figure 15d).
- Resilience: There is no significant difference in resilience before and after UV treatment, with a *p*-value of 0.8261 (Figure 15e).
- Toughness: There is a significant difference in toughness before and after UV treatment, with a *p*-value of 0.0090 (Figure 15f).

**Figure 16:**
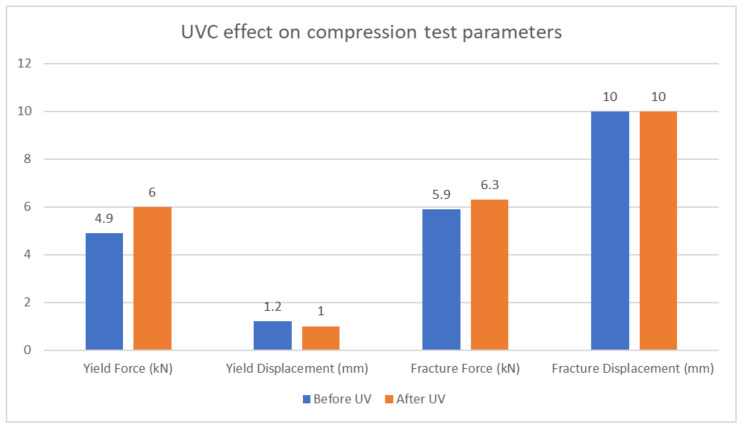
UVC effect on compression test parameters.

Table 15 and Figure 16 provide a clear representation of the effects of UVC treatment on the material’s yield stress, ultimate stress, and fracture stress. Here are the findings:

- Yield force: The yield force before UVC treatment is 4.9 kN, while after UVC treatment it increases to 6 kN. This significant increase indicates that the UVC treatment has a positive effect on the material’s ability to withstand applied forces. The material becomes stronger and more resistant to deformation under load.
- Fracture force: The fracture force before UVC treatment is 5.9 kN, and after UVC treatment, it increases to 6.3 kN. This substantial increase in fracture stress indicates that the UVC treatment enhances the material’s overall strength and its ability to resist breaking or fracturing under extreme loads.

**Table 15:** Parameters of compression specimens.

Average	Yield/Ultimate Force (kN)	Yield/Ultimate Displacement (mm)	Fracture Force (kN)	Fracture Displacement (mm)
Before UV	4.9	1.2	5.9	10
After UV	6	1	6.3	10

### 3.7. Energy Dispersive Spectroscopy (EDS) Analysis

The second method of validation involves the use of energy-dispersive X-ray spectroscopy (EDS) analysis. This technique was employed to examine the energy distribution within the tensile test specimens before and after UVC disinfection, with the goal of identifying and quantifying specific elements present within the surface of the samples. Each element possesses a unique atomic structure, which gives rise to distinct peaks on its electromagnetic emission spectrum.

**Figure 17:**
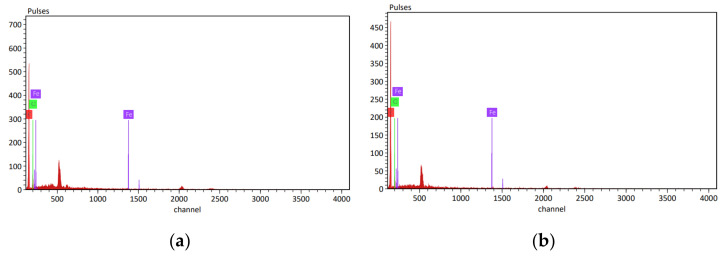
The spectrum of EDS. (**a**) The spectrum before treatment using UVC and (**b**) the spectrum after using UVC.

**Figure 18:**
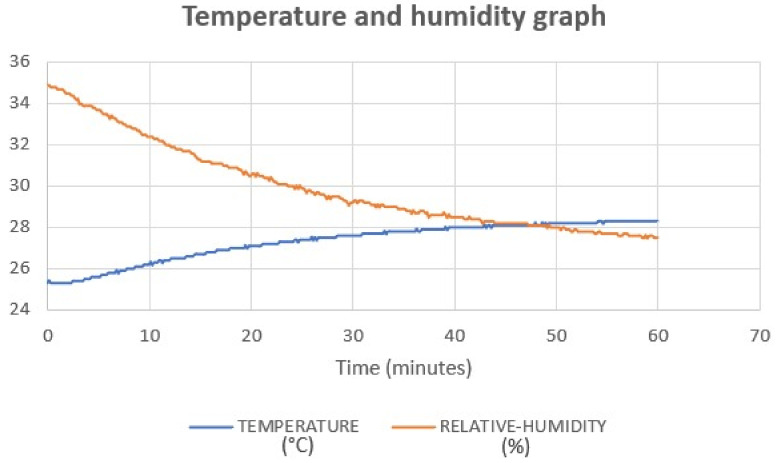
Temperature and humidity graph.

As depicted in Figure 17 and Table 16, the EDS analysis spectrum illustrates three major components: carbon (in red color), oxygen (in green color), and iron magnet (in violet color). The analysis revealed changes in element concentrations after UVC treatment, as follows:

- Carbon: The mass percentage of carbon increased from 71.69% before treatment to 78.56% after treatment, indicating an enhancement in hardness and strength. This suggests an improvement in the material’s hardenability, contributing to overall mechanical improvements.
- Oxygen: The mass percentage of oxygen decreased from 27.51% before treatment to 21.06% after treatment. This reduction could be attributed to UVC irradiation causing an increase in temperature during disinfection, which, in turn, leads to decreased oxygen content within the specimens. The temperature and humidity data logger data supports this finding, showing increased temperature and decreased humidity during UVC disinfection (Figure 18). This correlation between temperature and oxygen diffusion aligns with previous research [32].
- Iron magnet: There was a slight degradation in the iron magnet element after UVC treatment, with the mass percentage decreasing from 0.53% before treatment to 0.38% after treatment.

The EDS analysis results indicate that UVC disinfection positively influenced the chemical structure and hardenability of the material. The composite magnet material experienced an overall improvement in its properties due to the UVC disinfection process.

**Table 16:** Mass percentage before and after UVC irradiation.

Elements	Mass (%) Before UVC	Mass (%) After UVC
Carbon	71.69	78.56
Oxygen	27.51	21.06
Iron magnet	0.53	0.38

## 4. Discussion

The initial phase of this research revolved around meticulous material selection and the fabrication process of specimens. The meticulous execution of material selection, resin mixture preparation, and integration of magnetic components underscores the careful approach taken to ensure the viability and success of subsequent research stages. Precision in manufacturing and adherence to standardized dimensions further accentuate the commitment to accuracy and consistency in this study. This commitment to precision, combined with strict adherence to established guidelines, solidifies the reliability and credibility of the research findings.

The incorporation of specialized equipment, vigilant monitoring, and methodical arrangement underscores the methodical nature of the experimental setup, amplifying the accuracy and dependability of ensuing outcomes. Through the utilization of the provided equation, the study obtains a quantitative tool that aids strategic decision-making concerning exposure time, aligning seamlessly with desired objectives.

The application of energy-dispersive X-ray spectroscopy (EDS) serves as a robust means to unveil material’s surface composition, decipher constituent elements, and enhance our understanding of their chemical properties. The integration of advanced instruments like the Brucker xflash 6130 alongside SEM imagery exemplifies the careful approach taken to attain precise and comprehensive elemental analysis.

Additionally, the incorporation of visual and graphical representations such as stress–strain curves and force–displacement curves, related to tensile and compression test outcomes, in accordance with the exacting guidelines of ASTM D3039 and ASTM D3410, respectively, underscores the meticulous methodology of this experiment. These representations prove invaluable for comprehending material behavior under diverse conditions, playing a pivotal role in advancing our understanding of its response to external influences, as well as its mechanical properties and reactions to external forces.

Values derived from the force–displacement curve contribute substantially to understanding the material’s reaction to UVC treatment. They shed light on its capacity to endure forces, undergo deformation, and ultimately fracture, revealing essential insights into its mechanical characteristics and behavior.

Furthermore, the tabulated summaries of tensile and compression test results effectively capture the material’s mechanical responses and behaviors under varying conditions. These recorded values provide a comprehensive view of the forces and displacements experienced by material specimens during the tensile and compression tests. These data play a crucial role in interpreting the material’s reaction to applied stress and deformation, facilitating a detailed characterization of its mechanical properties. The methodical labeling and thorough analysis of these parameters underscore the rigorous methodology adopted in this study.

Quantitative assessments of resilience and toughness offer deeper insights into the material’s capability to withstand external forces and absorb energy without permanent deformation or fracturing. By evaluating these aspects, a holistic understanding of the material’s durability and energy absorption potential is achieved.

These quantifications encapsulate the influence of the disinfection process on the material’s mechanical attributes. The contrasts between pre-treatment and post-treatment values underscore the significance of UV irradiation in shaping the material’s response to external stressors.

Finally, the statistical analyses provide valuable insights into the effects of UV treatment on the material’s mechanical properties. The broad spectrum of parameters examined highlights the nuanced alterations induced by UV irradiation and contributes to a comprehensive grasp of the material’s behavior.

The study’s outcomes were highly compelling, demonstrating a significant and positive influence of UVC irradiation on the mechanical properties of the composite magnet material. In the tensile tests, the transition force exhibited a remarkable escalation from a pre-UVC exposure value of 0.41 kN to 0.58 kN after exposure. Similarly, the compression test results showcased a substantial enhancement in the yield force, with values increasing from 4.9 kN prior to UVC treatment to 6 kN following treatment. Noteworthy is the energy dispersive X-ray spectroscopy (EDS) analysis, which unveiled a marked rise in the carbon mass percentage. This percentage increased from 71.69% before UVC radiation exposure to 78.56% after exposure, indicating a clear improvement in material hardness. This observation further reinforces the promising potential of UVC irradiation as an effective disinfection method.

## 5. Conclusions

This study demonstrated the positive impact of utilizing UVC irradiation as a disinfection method for composite magnetic materials. This evaluation was carried out through two distinct approaches: mechanical testing involving both tensile and compression tests, and examination via EDS to assess the chemical composition of the material’s surface. The results obtained from the tensile and *t*-tests revealed significant differences in various mechanical parameters before and after UVC irradiation, including transition, ultimate, and fracture stress, together with ultimate strain, resilience, and toughness. However, no notable differences were observed between the transition and fracture strains. The tensile force–displacement curve displayed an increase in the transition force, transition displacement, ultimate force, ultimate displacement, and fracture force after UVC irradiation, with the exception of the fracture force, which decreased upon UV treatment. Similarly, the compression tests and associated *t*-tests indicated significant variations in the yield stress, yield strain, fracture stress, and toughness between the samples before and after UVC irradiation. However, no substantial differences were observed in the fracture strain and resilience. The compression force–displacement curve showed an increased yield force and fracture force after UVC treatment, whereas the yield displacement decreased, and the fracture displacement remained unchanged. Finally, the EDS analysis findings indicated improvements in the chemical structure and hardenability of the composite material, as a result of UVC irradiation during the disinfection process. Based on the collective insights gleaned from this study, the disinfection procedure utilizing UVC irradiation has an overall beneficial impact on composite magnetic material properties.

## Figures and Tables

**Table 9:** Mechanical properties of the untreated compression specimens (without UV).

Specimen Label	Yield Stress (N/mm^2^)	Yield Strain	Ultimate Stress (N/mm^2^)	Ultimate Strain	Fracture Stress (N/mm^2^)	Fracture Strain
B1	38	0.05	38	0.05	46	0.39
B2	39	0.05	39	0.05	48	0.39
B3	39	0.05	39	0.05	46	0.39
B4	37	0.05	37	0.05	46	0.39
B5	37	0.05	37	0.05	47	0.39
Average	38	0.05	38	0.05	47	0.39
±std	0.9	0	0.9	0	0.6	0

**Table 10:** Resilience and toughness for the five untreated compassion specimens (without UV).

Specimen Label	Resilience (J/m^3^)	Toughness (J/m^3^)
B1	0.69	14
B2	0.71	14
B3	0.71	14
B4	0.66	13
B5	0.77	14
Average	0.71	14
±std	0.04	0.23

**Table 11:** Forces and displacements of the untreated compression specimens (without UV).

Specimen Label	Yield Force (kN)	Yield Displacement (mm)	Ultimate Force (kN)	Ultimate Displacement (mm)	Fracture Force (kN)	Fracture Displacement (mm)
B1	4.8	1.2	4.8	1.2	5.9	10
B2	5	1.2	5	1.2	6	10
B3	4.9	1.2	4.9	1.2	5.9	10
B4	4.7	1.2	4.7	1.2	5.9	10
B5	5.1	1.3	5.1	1.3	5.8	10
Average	4.9	1.2	4.9	1.2	5.9	10
±std	0.15	0.03	0.15	0.03	0.08	0

**Table 12:** Mechanical properties of the treated compression specimens (with UV).

Specimen Label	Yield Stress (N/mm^2^)	Yield Strain	Ultimate Stress (N/mm^2^)	Ultimate Strain	Fracture Stress (N/mm^2^)	Fracture Strain
B6	46	0.04	46	0.04	49	0.39
B7	47	0.04	47	0.04	50	0.39
B8	49	0.04	49	0.04	52	0.39
B9	46	0.04	46	0.04	48	0.39
B10	48	0.04	48	0.04	51	0.39
Average	47	0.04	47	0.04	50	0.39
±std	1.3	0	1.3	0	1.7	0

**Table 13:** Resilience and toughness for the five treated compassion specimens (with UV).

Specimen Label	Resilience (J/m^3^)	Toughness (J/m^3^)
B6	0.69	14
B7	0.7	15
B8	0.73	15
B9	0.71	14
B10	0.73	15
Average	0.71	15
±std	0.02	0.45

**Table 14:** Forces and displacements of the treated compression specimens (with UV).

Specimen Label	Yield Force (kN)	Yield Displacement (mm)	Ultimate Force (kN)	Ultimate Displacement (mm)	Fracture Force (kN)	Fracture Displacement (mm)
B 6	5.8	1	5.8	1	6.2	10
B 7	5.9	1	5.9	1	6.4	10
B 8	6.1	1	6.1	1	6.5	10
B 9	6.2	1	6.2	1	6.5	10
B 10	5.9	1	5.9	1	6.1	10
Average	6	1	6	1	6.3	10
±std	0.16	0	0.16	0	0.22	0

## Data Availability

The data presented in this study are available on request from the corresponding author.
